# Robotic lobectomy has the greatest benefit in patients with marginal pulmonary function

**DOI:** 10.1186/s13019-018-0748-z

**Published:** 2018-06-05

**Authors:** Peter J. Kneuertz, Desmond M. D’Souza, Susan D. Moffatt-Bruce, Robert E. Merritt

**Affiliations:** Department of Surgery, Thoracic Surgery Division, The Ohio State University Wexner Medical Center, Doan Hall N846, 410 W 10th Avenue, Columbus, OH 43210 USA

**Keywords:** Robotic, Lobectomy, High risk, Pulmonary function, Outcomes

## Abstract

**Background:**

Patients with limited pulmonary function have a high risk for pulmonary complications following lobectomy. Robotic approach is currently the least invasive approach. We hypothesized that robotic lobectomy may be of particular benefit in high-risk patients.

**Methods:**

We reviewed our institutional Society of Thoracic Surgeons (STS) data on lobectomy patients from 2012 to 2017. Postoperative outcomes were compared between robotic and open lobectomy groups. High-risk patients were identified by pulmonary function test. Risk of pulmonary complication was assessed by binary logistic regression analysis.

**Results:**

A total of 599 patients underwent lobectomy by robotic (*n* = 287), or by open (*n* = 312) approach, including 189 high-risk patients. Robotic lobectomy patients had a lower rate of prolonged air leak (6% vs. 10%, *p* = 0.047), less atelectasis requiring bronchoscopy (6% vs. 16%, *p* = 0.02), pneumonia (3% vs. 8%, *p* = 0.01), and shorter length of stay (4 vs. 6 days, *p* = 0.001). Overall pulmonary complication rate was significantly lower after robotic lobectomy in high-risk patients (28% vs. 45%, *p* = 0.02), less in intermediate or low risk patients. No significant difference was seen relative to major complication rate (12% vs. 17%, *p* = 0.09). After multivariate analysis, when adjusting for age, gender, smoking history, FEV1, DLCO, cardiopulmonary comorbidities, and prior chest surgery, the robotic approach remained independently associated with decreased pulmonary complications (odds ratio 0.54, 95% confidence interval [0.34–0.85], *p* = 0.008).

**Conclusions:**

Robotic lobectomy has the potential to decrease the risk of postoperative pulmonary complication as compared with traditional open thoracotomy. In particular, patients with limited pulmonary function derive the most benefit from a robotic approach.

## Background

Posterolateral open thoracotomy has been the traditional approach to pulmonary lobectomy, which is associated with significant morbidity and a decrease in functional reserve capacity (FRC). Minimally invasive techniques using video-assisted thoracoscopic surgery (VATS) and more recently robotic assisted lobectomy have been developed to enhance recovery by decreasing complications, shorten length of stay and improve quality of life [1, 2]. Robotic lobectomy uses a completely port based approach, which is currently the least invasive technology. Despite the increased cost as compared with VATS, many surgeons have found value in the robotics due to improved three-dimensional visualization, increased freedom of instrument motion, and precise instrument movement with 3:1 motion scaling [3]. Nationally, the use of robotic lobectomy for lung cancer has tripled between 2010 and 2012 in the US from 3 to 9% [4]. In year 2015, more than 8600 pulmonary lobectomies have been performed robotically [5].

Although the overall robotic lobectomy experience is growing, less is known about the use of robotics in high-risk patients. As with any new technology, it is natural to select the easier cases in the early experience. Concerning anatomic lung resection in patients with intrinsic lung disease and poor pulmonary function, traditional thoughts may fear a higher incidence of air leaks, and possibly increased pain due to torqueing of the robotic ports. However, based on the benefit of minimally invasive lobectomy in patients with impaired pulmonary reserve in the VATS experience, we have been liberal in applying robotics in patients with increased pulmonary risk. We hypothesize, that robotic lobectomy may be of particular benefit in high-risk patients with marginal baseline pulmonary function. In this current study we aim to assess the postoperative outcomes of robotic lobectomy patients as compared with thoracotomy patients and to determine the relative impact of robotic approach on outcomes in high-risk patients undergoing lobectomy.

## Methods

This is a retrospective cohort study of patients who underwent lobectomy at a single institution. The study was approved by The Ohio State Institutional Review Board (Study#2018-C0021) and granted waiver of individual patient consent. The institutional Society of Thoracic Surgery (STS) general thoracic database was queried for patients who underwent lobectomy between January 1st 2012 and August 31st 2017. Included were all patients who underwent lobectomy by either robotic-assisted or open approach using thoracotomy. Patients who underwent, bronchoplastic, sleeve resection, or bilobectomy were excluded. Patients undergoing resection of Pancoast tumors or concomitant chest wall resection were also excluded.

The selection of surgical approach was at the discretion and experience of the surgeon. Robotic lobectomy was performed with a 4-arm technique with ports placed in line in the 8th intercostal space. We used the DaVinci Si robot until 2014 and thereafter the Xi model (Intuitive Surgical, Sunnyvale, CA). Additionally a 12 mm access port was used to for manual stapling, and since 2016 we have utilized the robotic controlled endo-wrist stapler. Stapled bronchial closure was performed in all patients.

Demographics, comorbidities, smoking status, use of induction therapy, lung cancer staging and prior thoracic surgery were reviewed. The definitions of postoperative events were categorized according to the STS definitions. The primary endpoint was pulmonary complication, as defined by the presence of any perioperative pulmonary complications, including air leak > 5 days, pneumonia, atelectasis requiring bronchoscopy, pleural effusion requiring drainage, pneumothorax requiring chest tube reinsertion, initial ventilator support > 48 h, bronchopleural fistula or tracheobronchial injury, respiratory failure, tracheostomy, acute respiratory distress syndrome (ARDS), or other pulmonary event (including pulmonary embolus). Respiratory failure was defined as impaired gas exchange, which requires reintubation and mechanical ventilation. According to the American College of Chest Physicians (ACCP) guidelines, preoperative pulmonary function tests were used to attribute surgical risk [6–8]. High risk was defined as preoperative forced expiratory volume in one second (FEV1) or diffusing capacity of the lung for carbon monoxide (DLCO) less than 60% predicted, as previously described [6]. Low risk were defined as patients with FEV1and DLCO > 80% [8], and intermediate risk patients as those with FEV1 or DLCO between 60 and 80%.

### Statistical analysis

Robotic and open lobectomy patients were compared using Chi-square/Fisher’s exact test for categorical variables, and Student’s T-test or Mann-Whitney U test for continuous variables based on presence of normal distribution. Univariate and multivariate binary logistic regression analysis was performed to test the association of clinical factors with pulmonary complications. Variables with *p* < 0.01 on univariate analysis were included in the multivariate model. Statistical significance was defined as a *p*-value ≤0.05. Data analysis was performed using SPSS 24.0 (LEAD Technologies, Inc., Chicago, IL) statistical software package.

## Results

A total of 599 patients underwent lobectomy by robotic (*n* = 287) or open approach (*n* = 312). Six patients who started with a robotic approach were converted to thoracotomy for limited visibility (*n* = 4) or restricted mobility (*n* = 2). Results were analyzed based on the approach in which the lobectomy was completed. Demographics and patient characteristics are summarized in Table 1. Patients who underwent robotic lobectomy were slightly older (mean age 65.3 ± 11.3 vs. 63.2 ± 10.1, *p* = 0.02), had better pulmonary function by spirometry (mean FEV1 82.4 ± 19.8 vs. 77.6 ± 19.7, *p* = 0.003), and had less frequent prior cardiothoracic surgery (14% vs. 20%, *p* = 0.04). Fewer patients in the robotic group underwent preoperative chemotherapy for clinical stage IIIa lung cancer (4% vs. 8%, *p* = 0.03). On final pathology, most patients had lung cancer on final pathology, with similar stage distribution between groups (Table 1).

**Table 1:** Baseline patient demographics and clinical characteristics of patients undergoing robotic and open lobectomy

	Robotic Lobectomy	Open Lobectomy	*P*-value
	(*n* = 287)	(*n* = 312)	
Age [years], mean ± SD	65.3 ± 11.3	63.2 ± 10.1	0.016
Male Gender	134 (47%)	157 (50%)	0.41
Zubrod Score			
0–1	270 (94%)	291 (93%)	0.67
2+	17 (6%)	21 (7%)	
Pack years, median (IQR)	40 (21–60)	40 (23–60)	0.51
Active smoker	91 (32%)	92 (30%)	0.59
FEV1 [% predicted], mean ± SD	82.4 ± 19.7	77.6 ± 19.7	0.003
DLCO [% predicted], mean ± SD	75.5 ± 22.8	74.5 ± 20.2	0.59
COPD	128 (45%)	118 (38%)	0.09
BMI > 30 kg/m^2^	88 (31%)	100 (32%)	0.80
Hypertension	180 (63%)	188 (60%)	0.56
Steroid use	9 (3%)	10 (3%)	0.96
Coronary artery disease	121 (42%)	132 (42%)	0.97
Peripheral vascular disease	29 (10%)	22 (7%)	0.19
Prior cardiothoracic surgery	40 (14%)	64 (20%)	0.040
Preoperative chemotherapy	11 (4%)	26 (8%)	0.027
Preoperative radiation therapy	13 (5%)	25 (8%)	0.10
Diabetes	45 (16%)	61 (20%)	0.22
Preoperative creatinine > 1.5 mg/dl	25 (4%)	13 (4%)	0.98
Lung cancer	240 (83%)	256 (82%)	0.61
Pathologic Stage (AJCC 7th ed.)			
Stage I-II	209 (87%)	220 (86%)	0.75
Stage III-IVa	31 (13%)	35 (14%)	
Laterality			
Left	176 (61%)	197 (63%)	0.65
Right	111 (39%)	115 (37%	

### Outcomes

Overall rate of pulmonary complication was lower following robotic as compared with open lobectomy (21% vs. 32%, *p* = 0.002), which included lower rates of prolonged air leak, atelectasis, pneumonia, and ARDS (Table 2). There were no intraoperative tracheobronchial injuries and one bronchopleural fistula (0.3%) in both groups. Median length of stay was two days shorter following robotic lobectomy (4 days vs. 6 days, *p* = 0.001). There was no significant difference in major complication rate or mortality (Table 2).

**Table 2:** Pulmonary complication and morbidity

Outcome	Robotic Lobectomy	Open Lobectomy	*P*-value
	(*n* = 287)	(*n* = 312)	
Pulmonary complication	60 (21%)	100 (32%)	0.002
Air leak (> 5 days)	16 (6%)	31 (10%)	0.047
Atelectasis (req. bronchoscopy)	18 (6%)	51 (16%)	0.001
Pleural effusion (req. drainage)	5 (2%)	9 (3%)	0.36
Pneumonia	9 (3%)	25 (8%)	0.010
Respiratory failure	22 (8%)	35 (11%)	0.14
ARDS	0	8 (2%)	0.031
Pneumothorax	12 (4%)	10 (3%)	0.52
Initial vent support > 48 h	0	2 (1%)	0.17
Tracheostomy	2 (1%)	8 (3%)	0.07
Other pulmonary event	6 (2%)	6 (2%)	1.00
Length of hospitalization [days], median (IQR)	4 (3–6)	6 (5–10)	0.001
Major Complications	34 (12%)	52 (17%)	0.09
Mortality	3 (1%)	5 (2%)	0.58

The regression analysis of predictors of pulmonary function is presented in Table 3. On univariate analysis, active smoking, increasing total pack year smoking history, low FEV1 and DLCO, coronary artery disease, peripheral vascular disease, chronic obstructive pulmonary disease (COPD) and thoracotomy were associated with pulmonary complications (Table 3). On multivariate analysis, robotic lobectomy was the independently associated with a decreased risk for pulmonary complications (odds ratio (OR) 0.53, 95% confidence interval (CI) 0.34–0.85], Table 3). Other predictive factors in the multivariate model were FEV1 and peripheral vascular disease (Table 3).

**Table 3:** Binary logistic regression analysis of factors associated with pulmonary complications

Characteristic	Univariate analysis			Multivariate analysis		
	Crude OR	95% CI	*p*-value	Adjusted OR	95% CI	*p*-value
Age	1.02	[0.99–1.03]	0.07	1.02	[0.99–1.05]	0.19
Male Gender	1.37	[0.95–1.97]	0.09	1.15	[0.73–1.82]	0.53
Active smoker	1.48	[1.01–2.17]	0.043	1.16	[0.72–1.85]	0.54
Pack Years (per year)	1.01	[1.00–1.02]	0.006	1.01	[0.96–1.01]	0.35
FEV1 (per % predicted)	0.97	[0.96–0.98]	< 0.001	0.97	0.96–0.98	< 0.001
DLCO (per % predicted)	0.99	[0.98–0.99]	0.006	1.00	[0.99–1.01]	0.60
Zubrod Score						
0–1	Reference					
2–5	1.46	[0.73–2.93]	0.28			
BMI						
20–30	Reference					
< 20	1.61	[0.85–3.07]	0.14			
> 30	0.89	[0.59–1.34]	0.58			
Steroid use	0.98	[0.35–2.76]	0.97			
Coronary artery disease	2.22	[1.51–3.14]	< 0.001	1.40	[0.87–2.23]	0.16
Peripheral vascular disease	2.69	[1.50–4.82]	0.001	1.99	[1.03–3.86]	0.042
Prior cardiothoracic surgery	1.67	[1.07–2.62]	0.026	1.62	[0.93–2.82]	0.088
Preoperative chemotherapy	1.53	[0.76–3.08]	0.24			
Preoperative radiation therapy	1.29	[0.64–2.62]	0.48			
Diabetes	1.23	[0.78–1.96]	0.37			
Last Cr > 1.5	1.87	[0.82–4.25]	0.14			
Last Hgb < 10	1.25	[0.52–2.85]	0.65			
COPD	2.42	[1.67–3.49]	< 0.001	1.62	[0.96–2.69]	0.052
Approach						
Open	Reference			Reference		
Robotic	0.56	[0.39–0.81]	0.002	0.54	[0.34–0.85]	0.008

### Comparison of high risk patients

**Fig. 1:**
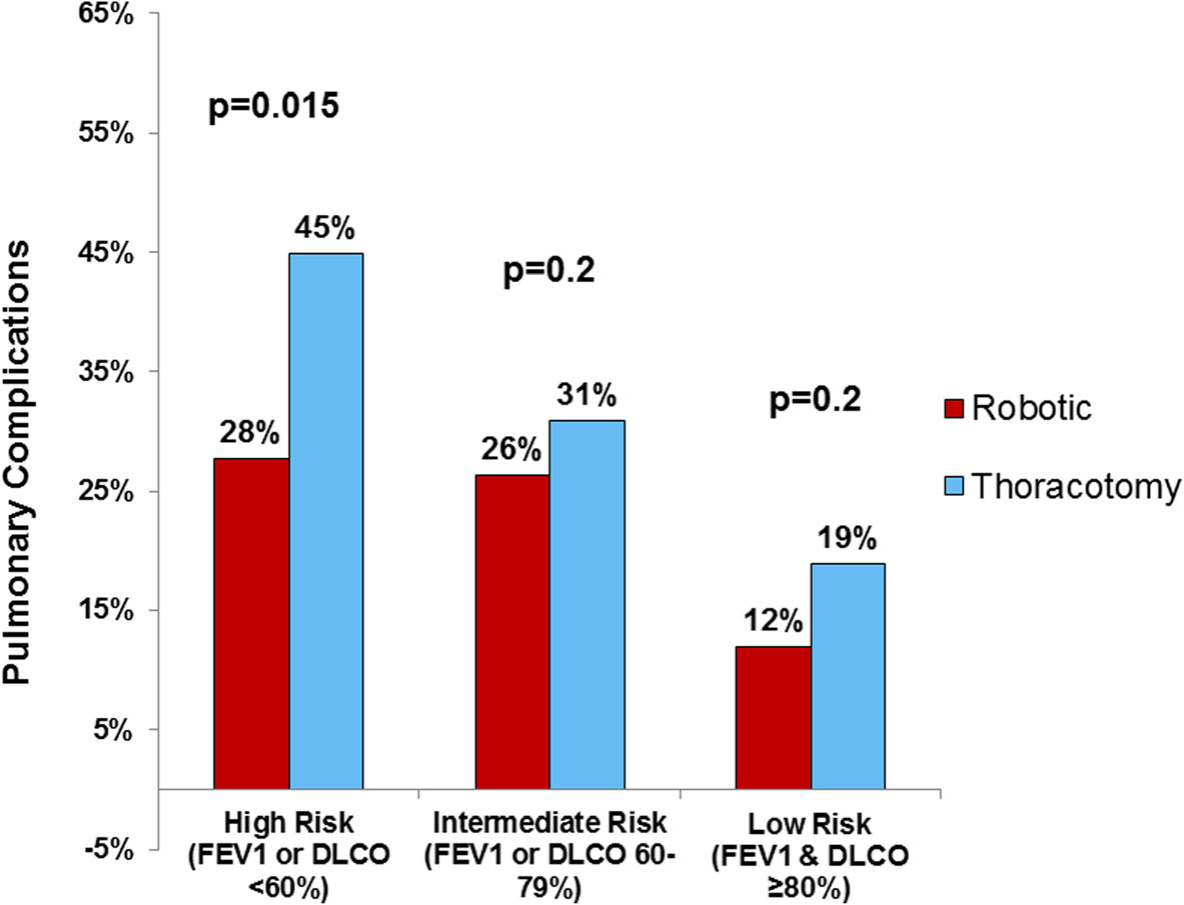
Pulmonary complications following lobectomy by preoperative risk group, comparing robotic-assisted and open thoracotomy approach

A subgroup analysis of 191 (32%) high-risk patients included 82 robotic and 107 open lobectomies. High-risk patients in the robotic group included more older patients > 75 years (22% vs. 11%, 0.05), more active smokers 46% vs. 31%, *p* = 0.04), and more patients with COPD (81% s. 54%, *p* < 0.001). There was no difference in lung cancer stage between robotic and thoracotomy groups in the high risk patients. High-risk patients undergoing robotic lobectomy were less likely to have any pulmonary complication (28% vs. 45%, *p* = 0.02). The difference in pulmonary complications between robotic and open lobectomy was greatest for high-risk patients, and less pronounced for with intermediate or intermediate or low risk patients (Fig. 1). Similarly to the entire cohort, robotic lobectomy in high-risk patients was associated with decreased rates of prolonged air leak, atelectasis and pneumonia (Table 4). In addition, none of the robotic lobotomy high-risk patients developed ARDS, needed a tracheostomy, or required initial vent support > 48 h (Table 4). Median length of stay following robotic lobectomy in high risk patients was 4 days and significantly shorted as compared with 8 days following open lobectomy (*p* < 0.001).

**Table 4:** Comparison of high-risk patients undergoing robotic- and open lobectomy

Characteristics	Robotic Lobectomy	Open Lobectomy	*p*-value
	(*n* = 82)	(*n* = 107)	
Age > 75 years	18 (22%)	12 (11%)	0.05
Male Gender	38 (46%)	55 (51%)	0.44
Zubrod Score			
0–1	78 (94%)	100 (93%)	0.88
2+	5 (6%)	7 (7%)	
Pack years, median (IQR)	50 (25–67)	40 (30–66)	0.035
Active smoker	38 (46%)	33 (31%)	0.035
COPD	67 (81%)	58 (54%)	< 0.001
FEV1 [% predicted], mean ± SD	67.0 ± 17.9	62.8 ± 18.0	0.10
DLCO [% predicted], mean ± SD	53.6 ± 15.7	57.8 ± 16.6	0.08
BMI > 30 kg/m^2^	20 (24%)	33 (31%)	0.47
Hypertension	60 (72%)	71 (66%)	0.38
Coronary artery disease	47 (57%)	52 (49%)	0.27
Peripheral vascular disease	11 (13%)	10 (9%)	0.47
Prior cardiothoracic surgery	13 (16%)	26 (24%)	0.15
Preop. Chemotherapy	5 (6%)	7 (7%)	0.88
Lung Cancer	69 (83%)	90 (84%)	0.86
Outcomes			
Pulmonary Complication	23 (28%)	48 (45%)	0.015
Air Leak (> 5 days)	4 (5%)	16 (15%)	0.024
Atelectasis (req. bronchoscopy)	6 (7%)	24 (22%)	0.005
Pleural effusion (req. drainage)	1 (1%)	5 (5%)	0.17
Pneumonia	3 (4%)	12 (11%)	0.05
Respiratory Failure	10 (12%)	13 (13%)	
ARDS	0	3 (3%)	0.12
Pneumothorax	3 (4%)	6 (6%)	0.52
Initial vent support > 48 h	0	2 (2%)	0.21
Tracheostomy	0	6 (6%)	0.028
Other pulmonary event	3 (4%)	1 (1%)	0.32
Length of hospitalization [days] Median (IQR)	4 (4–7)	8 (5–12)	< 0.001
Major Complications	16 (19%)	26 (24%)	0.41
Mortality	1 (1%)	2 (2%)	0.73

## Discussion

Pulmonary complications are the most common postoperative events following lung resection and can contribute to prolonged hospital length of stay and overall increased morbidity [9]. In this study of a large single institution dataset, we present that robotic lobectomy is associated with less pulmonary complication as compared with traditional open approach with thoracotomy in a high risk population. We show that while the risk of pulmonary complication increases in patients with worse pulmonary function regardless of approach, the robotic technique may serve to attenuate this risk independent of baseline pulmonary function. This study is important, because it includes a large set of patients with marginal pulmonary function, who were found to derive the greatest benefit from robotic lobectomy.

Minimally invasive thoracic surgery aims to expedite recovery by avoiding the chest wall trauma of a thoracotomy and the associated decrease in respiratory mechanics. Minimally invasive approach to lobectomy was first performed with VATS. Studies have shown the potential of the VATS approach to reduce blood loss, duration of chest tube drainage, postoperative pain, pulmonary complication and shorten overall length of hospitalization [1, 10]. Large population based studies have confirmed the benefit of the VATS approach with the reduction of pulmonary complications by 3–4% [6, 11]. Recent large population based studies comparing VATS and robotic approaches have shown comparable rates of overall complication rates and length of stay [4, 12]. Although our study does not directly compare robotic to VATS approach, the overall reduction of pulmonary complication rate of 11% compares favorably to the reported outcomes. This may be partly explained by the patient selection. In a recent study of the national STS database, Ceppa et al. showed that a VATS approach was associated with highest reduction in pulmonary complications for patients with impaired pulmonary function [6]. Our results confirm this finding, and show that the benefit of robotic approach is greatest for high-risk patients. High-risk patients are likely underrepresented in many current robotic lobectomy series, as surgeons may tend to select more straightforward cases when adopting new techniques [13]. One common concern has been the risk of postoperative air leak with a robotic approach, due to the lack of tactile feedback when manipulation diseased lungs, as well as the need for more dissection in the fissure, which could lead to increased risk of air leak. In this series we report a large number of high-risk patients undergoing robotic lobectomy, and demonstrate that the incidence of pulmonary complications can be significantly reduced using the robotic approach. Technical modifications that may have contributed to this result were cautious handling of the lung with the use of gauze rolls to avoid direct grasping. The risk of air leak may also be reduced in robotic lobectomy by improved visualization and precise dissection of the fissure with bipolar cautery, and the liberal use of staplers to divide the anterior and posterior fissure.

Lung cancer patients with poor pulmonary function present a challenge to thoracic surgeons. While lobectomy remains the best treatment for early stage lung cancer, alternative options are often considered for high-risk patients. In addition to sublobar resection, high-risk patients are also increasingly considered for stereotactic radiation (SBRT) or ablation [14]. Crabtree et al. reported a comparison of two prospective clinical trials of high-risk patients with stage I lung cancer using sublobar resection (ACOSOG Z4032) and SBRT (RTOG 0236), and shown no difference in early mobidity in appropriately matched patients [15]. Selection criteria for the ACOSOG Z4032 and the currently enrolling JoLT Stablemates (former Z4099), which is directly comparing sublobar resection with SBRT, define high-risk patients by using preoperative pulmonary function tests as major criteria and age and cardiovascular comorbidities as minor criteria [16]. However, there remains significant debate on the appropriate risk stratification. Several studies have questioned these empiric ACOSOG criteria by showing acceptable outcomes following lobectomy in so called high-risk patients [17–19]. A recent study by Taylor et al. examined high-risk patients undergoing VATS lobectomy and found similar early morbidity to normal risk patients and a 30-day mortality < 1% in carefully selected patients who fit the mentioned trial criteria [19]. Our results in lobectomy patients confirm that lobectomy can be safely performed in selected high-risk patients with marginal pulmonary function, and that the surgical approach should be weighed when determining if patients are appropriate for lobectomy to ensure optimal oncologic therapy for high-risk patients.

We do acknowledge several limitations of our study. Given the retrospective nature of this study, we cannot exclude that results have been biased by selection and confounding of unmeasured factors including surgeon preference of technique and experience. However, for the purpose of comparing pulmonary outcomes we have risk-stratified patients based on preoperative pulmonary function, and specifically compared the subgroup of high-risk patients. High-risk patients in the robotic group actually included older and sicker patients, as this series reflects a liberal application of robotics for higher risk patients beyond the initial learning curve. The large number of patients in this study allowed for rigorous multivariate modeling of pulmonary complication to control for confounding. In this study we were unable to compare the robotic approach to the VATS approach due the practice pattern at our institution, which should be inbestigated in a future study. Furthermore, the study is limited to comparison of perioperative outcomes and does not examine differences in pain, dyspnea, and health related quality of life. Postoperative pain may be a significant factor in reducing postoperative pulmonary morbidity and shorten recovery. Patient reported outcomes, cost, and long term outcomes are important topics for future studies.

## Conclusions

Robotic lobectomy may decrease risk of pulmonary complications as compared with traditional thoracotomy. Patients with poor pulmonary function have the potential to derive the most benefit from a robotic approach. Therefore, robotically assisted thoracic approaches should be considered when selecting high-risk patients requiring lobectomy.

## Abbreviations

ACCP: American College of Chest Physicians
AJCC: American Joint Committee on Cancer
ARDS: Acute respiratory distress syndrome
BMI: Body mass index
CI: Confidence Interval
CR: Serum Creatinine level
COPD: Chronic obstructive pulmonary disease
DLCO: Diffusing capacity of the lung for carbon monoxide
FEV1: Forced expiratory volume in one second
FRC: Functional reserve capacity
Hgb: Hemoglobin level
IQR: Inter quartile range
OR: Odds ratio
SD: Standard deviation
STS: Society of Thoracic Surgeons
VATS: Video-assisted thoracoscopic surgery

## References

[1] Scott WJ, Allen MS, Darling G (2010). Video-assisted thoracic surgery versus open lobectomy for lung cancer: a secondary analysis of data from the american college of surgeons oncology group z0030 randomized clinical trial. J Thorac Cardiovasc Surg.

[2] Li WW, Lee TW, Lam SS (2002). Quality of life following lung cancer resection: video-assisted thoracic surgery vs thoracotomy. Chest.

[3] Ramadan OI, Wei B, Cerfolio RJ (2017). Robotic surgery for lung resections-total port approach: advantages and disadvantages. J Vis Surg.

[4] Rajaram R, Mohanty S, Bentrem DJ (2017). Nationwide assessment of robotic lobectomy for non-small cell lung cancer. Ann Thorac Surg.

[5] Wei B, Cerfolio RJ (2017). Robotic lobectomy and segmentectomy: Technical details and results. Surg Clin North Am.

[6] Ceppa DP, Kosinski AS, Berry MF (2012). Thoracoscopic lobectomy has increasing benefit in patients with poor pulmonary function: a society of thoracic surgeons database analysis. Ann Surg.

[7] Burt BM, Kosinski AS, Shrager JB, Onaitis MW, Weigel T (2014). Thoracoscopic lobectomy is associated with acceptable morbidity and mortality in patients with predicted postoperative forced expiratory volume in 1 second or diffusing capacity for carbon monoxide less than 40% of normal. J Thorac Cardiovasc Surg.

[8] Brunelli A, Kim AW, Berger KI, Addrizzo-Harris DJ (2013). Physiologic evaluation of the patient with lung cancer being considered for resectional surgery: diagnosis and management of lung cancer, 3rd ed: American college of chest physicians evidence-based clinical practice guidelines. Chest.

[9] Kozower BD, O'Brien SM, Kosinski AS (2016). The society of thoracic surgeons composite score for rating program performance for lobectomy for lung cancer. Ann Thorac Surg.

[10] Oparka J, Yan TD, Ryan E, Dunning J (2013). Does video-assisted thoracic surgery provide a safe alternative to conventional techniques in patients with limited pulmonary function who are otherwise suitable for lung resection?. Interact Cardiovasc Thorac Surg.

[11] Paul S, Altorki NK, Sheng S (2010). Thoracoscopic lobectomy is associated with lower morbidity than open lobectomy: a propensity-matched analysis from the sts database. J Thorac Cardiovasc Surg.

[12] Kent M, Wang T, Whyte R, Curran T, Flores R, Gangadharan S (2014). Open, video-assisted thoracic surgery, and robotic lobectomy: review of a national database. Ann Thorac Surg.

[13] Mahieu J, Rinieri P, Bubenheim M (2016). Robot-assisted thoracoscopic surgery versus video-assisted thoracoscopic surgery for lung lobectomy: can a robotic approach improve short-term outcomes and operative safety?. Thorac Cardiovasc Surg.

[14] McMurry TL, Shah PM, Samson P, Robinson CG, Kozower BD (2017). Treatment of stage i non-small cell lung cancer: What's trending?. J Thorac Cardiovasc Surg.

[15] Crabtree T, Puri V, Timmerman R (2013). Treatment of stage i lung cancer in high-risk and inoperable patients: comparison of prospective clinical trials using stereotactic body radiotherapy (rtog 0236), sublobar resection (acosog z4032), and radiofrequency ablation (acosog z4033). J Thorac Cardiovasc Surg.

[16] Fernando HC, Timmerman R (2012). American college of surgeons oncology group z4099/radiation therapy oncology group 1021: a randomized study of sublobar resection compared with stereotactic body radiotherapy for high-risk stage i non-small cell lung cancer. J Thorac Cardiovasc Surg.

[17] Sancheti MS, Melvan JN, Medbery RL (2016). Outcomes after surgery in high-risk patients with early stage lung cancer. Ann Thorac Surg.

[18] Donahoe LL, de Valence M, Atenafu EG (2017). High risk for thoracotomy but not thoracoscopic lobectomy. Ann Thorac Surg.

[19] Taylor MD, LaPar DJ, Isbell JM, Kozower BD, Lau CL, Jones DR (2014). Marginal pulmonary function should not preclude lobectomy in selected patients with non-small cell lung cancer. J Thorac Cardiovasc Surg.

